# Anatomical Researches on the Thymus Gland in Man and Animals

**Published:** 1818-05

**Authors:** 


*Anatomische Untersuchungen der Thymus in Menschen und Thieren;* von SAMUEL CHRETIEN LUCAE, &c. (Anatomical Researches on the Thymus Gland in Man and Animals. By S. C. LUCAE). Quarto, pp. 55. Frankfort-on-the-Main, 1811.

ANATOMY has, of late years, been cultivated with such ardour and success, that the science now occupies a post, very far advanced, on the road to perfection. Yet there are still some organs of the animal œconomy, which have hitherto defied every effort to unravel the mysteries of their structure: and the physiologist, foiled and bewildered in his researches, is compelled to observe a humiliating silence, or resort to the dangerous employment of loose analogy, or the more unphilosophical mean of rash conjecture, respecting the functions which they are destined to perform.

Among these organs, the thymus holds a conspicuous station; for, although the existence of this gland has been known from a very early period of anatomical history, its intimate structure, the peculiar life and office with which it is endowed, yet lie involved in profound obscurity.

Rufus, of Ephesus, appears to have been the first writer by whom the thymus has been distinctly mentioned. A more detailed account of it may be found in the works of Galen, who ascribed to it the office of fixing the inferior vena cava, and invariably retaining it in the same situation with respect to the heart. This hypothesis, of which the discovery of the lymphatic system at length effected the subversion, prevailed in the schools till about the middle of the seventeenth century. At that period, Warton, not content with remaining the servile copyist of the celebrated physician Pergamus, interrogated Nature on this subject for himself. His example failed not to influence others; and the monographs of Metzger and Verheyen, and the controversial writings between the latter and Bidloo, resulted from the impulse thus communicated to talent. But all these authors were led astray from the sober but secure track which alone philosophy prescribes, in the vain pursuit of physiological theories. Haller and Duverney, however, followed a different route. Throwing aside all arbitrary explanations, they directed a particular attention to the structure of the thymus gland; without a knowledge of which, the impracticability of deciding upon the functions of an organ is at once obvious. Yet, valuable as were, in some respects, the anatomical details presented by these masterly writers, little light was reflected by them upon the obscurity of the subject: and much remained still to be explored and determined, ere we could hope to arrive at a satisfactory solution of this interesting and arduous problem in physiology.

In giving his observations to the public, Dr. Lucae has not the presumption to suppose that he is enabled to clear away all the difficulties which obstruct the path of this inquiry. He merely professes to develope some novel views, and offer some useful directions, to the physiologist in his researches upon the structure and functions of the thymus gland.

He commences by the attempt to controvert an erroneous notion, which has crept into most of the modern works on anatomy, respecting the size of the thymus at different periods of life. Many writers assert that the thymus presents, in an absolute manner, and not merely in relation to the respective differences of stature, a greater volume in the infant than in the adult. Dr. Lucae has drawn from conviction an opposite inference; and affirms, that the growth of the organ continues till a certain period after birth.

In the new-born child, the thymus extends from the superior extremity of the sternum, and often even from the lateral borders of the thyroid gland, to the point of insertion of the cartilages of the sixth ribs. When we reflect, that thus its inferior extremity reaches nearly to the level of the anterior attachments of the diaphragm, it is evident that the gland fills nearly the whole cavity of the mediastinum. Dr. Lucae has found it of this relative length, in all the subjects of an earlier age, whose bodies he has had occasion to inspect, and even in a child of five years. He moreover thinks, that the relation which exists between the depth of the thorax, and the length of the thymus gland, ceases somewhat later in animals than in man. This, at least, he has reason to believe, from repeated observation, is the case in the dog, the cat, the rabbit, and the squirrel.

That which the thymus acquires in length during the earlier period after birth, it seems to lose in thickness: for, in all the bodies of men and animals which it has fallen to his lot to examine, Dr. Lucae has invariably found the gland thinner in proportion as the age of the subject was more advanced.

Previously to the time of Dr. Lucae, similar observations had been made by Verheyen. Santorini and Meckel had also represented the thymus as frequently possessing greater volume in aged persons than in the fœtus. By some it will, perhaps, be regretted that Dr. Lucae has not endeavoured to fix, with something like precision, the period at which the organ commonly ceases to increase in bulk; nor studied the changes which occur in its intimate structure after birth. Notwithstanding the air of novelty with which he invests his explanation of the gradual diminution of the gland, it is, in fact, destitute of originality. Professor Portal has broached opinions nearly, if not precisely similar, on the subject. This successive decrease of volume is referred to the pressure exercised upon the thymus by the pulmonary organs:—with what degree of probability, we are incompetent accurately to determine. The authors and partizans of this hypothesis make the chief strength of their argument for its correctness to repose upon the fact that this decrease of the gland takes place from below upward, while the lungs dilate more at their base than at their summit. The application, however, of mechanical principles, to illustrations of the science of physiology, we are accustomed to view with a very sceptical, or at least a suspicious eye.

That the reader may be enabled to appreciate for himself the value of Dr. Lucae's labours, we shall now present to him a concise sketch of the observations made by the German physiologist upon the structure of the thymus gland.

On microscopically inspecting a slice of the thymus of a calf, Dr. Lucae saw that its lobules were formed externally by a whitish pultaceous substance, in the centre of which a brown spot was perceptible. From this spot, which was penetrated in every direction by very delicate vessels filled with blood, a yellowish, viscous fluid oozed, on slight compression. On the surface of the whitish substance were numerous minute red points, surrounded by a net-work of vessels. Here and there, these vessels seemed to unite the red points of two lobules placed in contiguity. Much irregularity existed in the distribution of the red points among the lobules: but they evidently were not formed by the diameter of a divided vessel. They exhibited rather the appearance of small groupes of interwoven blood-vessels, which communicated together by intermediate anastomoses.

After two hours' maceration in water, and subsequent washing of another slice, it became evident, without the aid of a microscope, that the supposed brown spot in each lobule of the gland, was, in fact, a small cavity filled with a yellow viscous fluid. The figure and size of this cavity invariably corresponded with those of its lobule. In other slices, dried, and held up against the light, the red points and vessels of the lobules were perceptible by the naked eye. Reiterated experiments of this kind on the thymus of the calf afforded similar results. But, on examining in like manner, the thymus of new-born children, Dr. Lucae detected only some slight traces of this structure, without being able to distinguish them clearly, on account of the tenuity of the parts.

In some thin slices of the thymus of a young rat, which had been macerated for an hour in water, were discerned, on holding it against the light, lobules somewhat resembling minute transparent vesicles, and less pellucid at the circumference than the centre.

A portion of the border of the thymus of a calf, after being macerated in water, and then laid on paper and turned, was examined against the light. The whole border seemed to be formed by a yellowish uniform transparent mass; and, at the distance of about one line from it, was seen a substance less pellucid, composed of small rounded bodies, which, in some degree, resembled raisin-stones. The microscope shewed that these minute bodies were formed by a congeries of infinitely fine and convoluted red vessels. They were every where surrounded by an homogeneous, yellowish, transparent, and semi-fluid mass, which was particularly abundant in those places where the limits between two lobules might be distinguished on the surface of the gland by the grooves of its external membrane.

The ensuing observations constitute the result of Dr. Lucae's latest researches on the intimate structure of the thymus:—On dividing the substance of this organ in the calf, he discovered a small cavity, more or less strongly marked, in each lobe. The form of this cavity invariably corresponded, although in no certain degree, with that of the lobule in the centre of which it was situated. But both cavity and lobule were alike destitute of regular shape and symmetry. When one of the cavities has been opened, if the point of a knife be introduced, in order to dilate the orifice and slit the lobule, the instrument may be observed, as soon as it comes in contact with the parietes of the sac, to experience a resistance, which requires the employment of a slight effort in penetrating the substance of the lobule. From this circumstance, it may be presumed that the internal surface of each cavity is lined by a particular membrane: and this conjecture derives farther support from the result of microscopical observation. The parietes themselves are formed by the grains of the gland closely compressed together. Each grain, moreover, appears to contribute to the formation of a cavity; and each of them, which is visible on the surface of the lobule, to extend to the interior of such cavity.

On examining, with a microscope, slices of the thymus gland of the calf, which for three years had been preserved in alcohol, the internal surface of the parietes of the cavities was seen to have become rough and unequal from a number of minute elevations. Upon some of the most considerable of these eminences were remarked small points, distinguishable by their grey colour from the rest of the surface, the general tint of which was white. Soon afterwards, these points were ascertained to be minute rounded orifices; into the interior of which, a fine pig's bristle might be introduced to the depth of a line, without, however, the possibility of ascertaining the distance to which the orifice extended. Attentive observation of several of these cavities induced the conviction that each of them contained from one to four of the small orifices in question.

In a recent state, each cavity contains a slightly viscous fluid resembling yellow and ropy pus. Anatomists have long been aware of the existence of this fluid; but none of them seemed to have entertained the faintest notion of the precise situation in which it is found. Haller was the first who remarked that the very substance of the thymus constituted its seat; and that division of the gland was necessary in order to obtain it. The fluid, from its slightly yellow colour, resembles pus rather than milk. Whether the tinge, which it exhibits, be referrible or not to an admixture of blood, Dr. Lucae declares himself unable to decide.

By Bartholine, de Graaf, Duverney, and Blumenbach, a great cavity in the interior of the thymus has been spoken of. But neither in man nor in animals has Dr. Lucae been able to discover it. He conjectures that the cavity in question may have been formed by these anatomists in dividing the substance of the organ: "for the cellular structure which it contains is so delicate, that, in making an incision, the viscidity of the fluid is sufficient to cause to adhere to the scalpel whole lobules or grains, which are thus displaced with violence from their natural position."

From all the observations which he has accumulated and recorded on the structure of the thymus gland, Dr. Lucae considers that the following general inferences may be deduced:—

1st. The thymus secretes a fluid corresponding to itself in colour. This circumstance, he adds, is met with in every part of the animal economy, destined to perform the office of secretion; and nothing more is wanting to prove that the thymus belongs to the class of secretory organs.

2d. Each lobule contains a cavity in its interior; which is, in some sort, the receptacle of the secreted fluid.

3d. The secretory apparatus is distributed in the circumference of this receptacle.

4th. Each grain contains a cluster of interwoven vessels, forming part of the secretory apparatus.

5th. As there exists a certain relation between the size of the cavities or receptacles, and the volume of the lobules; as the magnitude of the latter is determined by the number of grains which enter into their composition; and as each grain encloses a cluster of vessels, composing an integral part of the secretory apparatus, we may conclude that a certain relation also obtains between the capacity of the receptacle and the quantity of secreted fluid.

6th. It remains undecided, to the present day, whether the secreted fluid, considered in each lobule in particular, possesses an analogy with that of the mucous or the serous organs, or with that of the conglomerate glands; whether, consequently, the cavities of the lobules are mucous follicles or serous reservoirs; or, lastly, terminations of excretory ducts, after the manner of the gall-bladder and vesiculæ seminales. Upon the illegitimacy, and indeed obvious incorrectness, of the first in this series of inferences, we need not pause to offer any comment.

From the preceding abstract—all that our time and limits allow us to present—the reader will be enabled to determine for himself the character of Dr. Lucae's Treatise, and estimate its value. That the subject is not exhausted so long as the functions of the thymus remain undiscovered, every one must be aware. Yet, defective as are the labours of the German physiologist, in this as in some other respects, they must not be altogether slighted and condemned, as destitute of merit and unworthy of courteous reception. To fix previously fluctuating and unsettled opinions, and erect them as land-marks whereby to regulate its step, and to clear the obstructing masses of error from the road, are objects of high importance in all the excursions and enterprizes of the human mind. Efforts like these constitute the Pioneers of Science: although not indicative of her exact progress, they unequivocally announce her approach, and direct and facilitate her line of march. Nor is physiology, in our opinion, triflingly indebted to Dr. Lucae, for having given stability to the inference that the thymus, though principally formed for, exercises not its functions exclusively in, the fœtal system; and by unravelling the mysterious mechanism of its structure, assigned finally to the gland the situation, if not the character, among the secretory organs, to which it is entitled.

